# An insight into the swine-influenza A (H1N1) virus infection in humans

**DOI:** 10.4103/0970-2113.76299

**Published:** 2011

**Authors:** Girish L. Dandagi, Sujata M. Byahatti

**Affiliations:** *Department of Pulmonary Medicine, KLE’S Jawaharlal Nehru Medical College and Research Centre, Belgaum, Karnataka, India*; 1 *Department of Oral Medicine and Radiology, Maratha Mandals N.G. Halgekar Institute of Dental Sciences and Reaserch Centre, Belgaum, Karnataka, India*

**Keywords:** H1N1 influenza, pig flue, swine flu, swine influenza in humans, H1N1, India, vaccine

## Abstract

WHO declares on June 11, 2009, that H1N1 (Swine-influenza A) is pandemic. There have been nearly 30,000 confirmed H1N1 cases across 74 countries. The reports have shown sharp increase in the number of infections reported in recent days from Chile, Japan, and the UK, and other parts of the world, with the most dramatic increase recorded in Australia where more than 1200 cases were reported in a very short duration. As per the latest report of the Ministry of Health and Family Welfare, death from swine flu has reached to 1235. Around 12,3397 people have been tested in India as on February 1, 2010. In India, 23.3% of people who have tested for swine flu are found suffering from swine flu. Also around 4% of people who have tested positive for swine flu have died and could not be saved in India. *The New York Times* has reported that this is the first flu for being pandemic in the last 41 years. This article enlightens the brief review about the swine influenza virus, its modes of spread, and prevention measures. The aim of this article is to bring awareness in general and know the consequences of the infection.

## INTRODUCTION

Swine influenza is a highly contagious acute respiratory disease of pigs,[1] caused by one of the several strains of swine influenza A. The virus is spread among pigs by aerosols, through direct and indirect contact, and also by asymptomatic carrier pigs. Swine influenza seen predominantly in the mid-western United States (and occasionally in other states), Mexico, Canada, South America, Europe (including UK, Sweden, and Italy), Kenya, Mainland China, Taiwan, Japan, and other parts of eastern Asia and in various parts of India. In humans, the symptoms of swine flu are similar to those of influenza namely chills, fever, sore throat, muscle pains, severe headache, coughing, weakness, and general discomfort. WHO[2] says that no previous pandemic disease has been detected so early or watched so closely, in real-time, right at the very beginning. The world can now reap the benefits of investments, over the last 5 years, in pandemic preparedness.

## VIROLOGY

On April 15 and April 17, 2009, the Centers for Disease Control and Prevention[3] (CDC) identified two cases of human infection with a swine-origin influenza A (H1N1) virus. The World Organization for Animal Health reports[3] that Swine Influenza strain has not been isolated in pigs.[4] This strain can be transmitted from human to human[5] and causes the normal symptoms of influenza.[6] Basically these viruses causing pig’s flue are classified as influenza A, B, and C. Transmission mainly occurs between pigs and pigs and humans. The viruses are 80–120 nm in diameter.[7] Of the three genera of influenza viruses that cause human flu, two also cause influenza in pigs, with influenza virus A being common in pigs and influenza virus C being rare.[8] Influenza virus B has not been reported in pigs. Within influenza virus A and C, the strains found in pigs and humans are largely distinct, although due to reassortment there have been transfers of genes among strains crossing swine, avian, and human species boundaries.

## EPIDEMIOLOGY

Swine-origin influenza A (H1N1) virus in humans have been identified in swine in the United States since 1998,[9,10] and 12 cases of human infection with such viruses were identified in the United States from 2005 through 2009.[11] As of May 5, 2009, a total of 642 cases of human infection with a swine-origin influenza A (H1N1) virus have been identified in the United States, and other additional cases have been identified in Mexico, Canada, and South-East Asia.[3] On April 25, the WHO[12] declared a public health emergency of international concern, and on April 26, the United States declared a public health emergency. On April 29, the WHO raised the pandemic influenza phase from 4 to 5, indicating that human-to-human transmission of the virus was occurring in at least two countries in one WHO region. The observation[12] that 60% of patients were 18 years of age or younger suggests that children and young adults may be more susceptible to swine-origin influenza A infection than are older persons or that because of differences in social networks, transmission to older persons has been delayed. It is also possible that elderly persons may have some level of cross-protection against swine-origin influenza A infection from preexisting antibodies against other influenza A (H1N1) viruses, as suggested by serologic studies of the 1976 swine influenza vaccine.[13,14] A potential case-ascertainment bias may also exist, with more young people being tested as part of outbreaks of swine-origin influenza A infection in schools[15] and fewer older persons being tested for influenza. However, the epidemic is evolving rapidly, and the number of confirmed cases is an underestimate of the number of cases that have occurred. The age of patients with confirmed swine-origin influenza A[12] infection ranged from 3 months to 81 years. A total of 40% of patients were between the ages of 10 and 18 years, and only 5% of patients were 51 years of age or older. Among the patients for whom clinical information was available, the most common symptoms were fever (94%), cough (92%), and sore throat (66%). In addition, 25% of patients had diarrhea and 25% had vomiting. As per the latest report of the Ministry of Health and Family Welfare,[16] death from swine flu has reached to 1235. Around 123,397 people have been tested in India as on February 1, 2010. In India, 23.3% people who have tested for swine flu are found suffering from swine flu. Also around 4% people who have tested positive for swine flu have died and could not be saved in India. Maharashtra with 317 deaths has seen maximum deaths from swine flu in India. Gujarat also has 242 deaths from swine flu till February 1, 2010. Interestingly Gujarat is the only state where Chief Minister has been tested positive for swine flu. Other Indian states suffering from are Rajasthan (176 deaths), Karnataka (141 deaths), and Delhi (93 deaths).Delhi has the highest number of patients who are tested positive for swine flu, with 9,652 people tested positive. Maharashtra has 5,116 cases of swine flu affected patients and Rajasthan has 2,101 patients who were tested positive for swine flu till February 1, 2010. The states which are severely affected by swine flu have high exposure to foreigners. Also people from these states make more foreign visits. However the trend is changing now. On February 1, 2010, from the 51 patients who tested positive for swine flu, only one has traveled outside India and remaining 50 have infected in the country only.

## PATHOGENESIS

Swine influenza, also called swine flu, hog flu, and pig flu, refers to influenza caused by those strains of influenza virus,[1] called swine influenza virus (swine influenza virus), that usually infect pigs. People who work with pigs, especially people with intense exposures, are at increased risk of catching swine flu. In the mid-20^th^ century, identification of influenza subtypes became possible; this allows accurate diagnosis of transmission to humans. Since then, 50 confirmed transmissions have been recorded. Rarely, these strains of swine flu can pass from human to human. The 2009 H1N1 virus is not zoonotic swine flu, as it is not transmitted from pigs to humans, but from one person to person. In the United States, CDC[1] advised physicians to “consider swine influenza infection in the differential diagnosis of patients with acute febrile respiratory illness who have either been in contact with persons with confirmed swine flu, or who were in one of the five U.S. states that have reported swine flu cases or in Mexico during the 7 days preceding their illness onset.” The modes of transmission of influenza viruses in humans, including swine influenza, are not known but are thought to occur mainly through the dissemination of large droplets and possibly small-particle droplet nuclei[17] expelled when an infected person coughs. There is also potential for transmission through contact with fomites that are contaminated with respiratory or gastrointestinal material.[18,19] Since many patients with swine influenza infection have had diarrhea, the potential for fecal viral shedding and subsequent fecal–oral transmission should be considered and investigated. The incubation period for swine influenza infection appears to range from 2 to 7 days; however, additional information is needed. On the basis of data regarding viral shedding from studies of seasonal influenza, most patients with swine influenza infection might shed virus from 1 day before the onset of symptoms through 5 to 7 days after the onset of symptoms or until symptoms resolve; in young children and in immunocompromised or severely ill patients, the infectious period might be longer.[20] The clinical spectrum of novel swine influenza infection is both self-limited illness and in severe outcomes, it can lead to respiratory failure and death, which have been observed among identified patients with a wide clinical spectrum similar to that seen among persons infected with earlier strains of swine-origin influenza viruses[11] and seasonal influenza viruses.[21] The severe illness and deaths associated with seasonal influenza epidemics are in large part the result of secondary complications, including primary viral pneumonia, secondary bacterial pneumonia (particularly with group A *Streptococcus, Staphylococcus aureus*, and *Strep. pneumoniae*),[22–24] and exacerbations of underlying chronic conditions.[25] These same complications may occur with swine influenza infection. Patients who are at highest risk for severe complications of swine influenza infection are children under the age of 5 years, adults 65 years of age or older, children and adults of any age with underlying chronic medical conditions, and pregnant women.[26,27]

## CLINICAL FEATURES

According to CDC,[1] in humans the symptoms include fever, cough, sore throat, body aches, headache, chills, and fatigue. The 2009 outbreak has shown an increased percentage of patients reporting diarrhea and vomiting. It has been noted that around 2% of infected individuals[12] in some countries have developed severe disease, and that this has occurred most commonly in those aged 30–50 years. Many of these severe cases have involved other chronic health conditions; however, between a third and half of the serious cases have occurred in otherwise healthy, young-to-middle-aged people. Overall, the majority of infections have occurred in individuals aged younger than 25 years.

## LABORATORY DIAGNOSIS

A diagnosis of confirmed swine flu requires laboratory testing of a respiratory sample (a simple nose and throat swab). The CDC has developed a Swine Influenza Virus Real-Time PCR Detection Panel.[28] Among the various diagnostic tests, Real-Time PCR, Nucleotide Sequencing, and phylogenetic analyses were used.

### Real-time PCR

The CDC has developed a real-time PCR assay to detect seasonal influenza A, B, H1, H3, and avian H5 serotypes. Primers and probes specific for swine influenza A (H1 and H3 subtypes) were recently developed and tested for use in a modified version of this assay for the detection of human infection with swine influenza viruses.

### Nucleotide sequencing and phylogenetic analysis

Amplicons for gene sequencing were generated by reverse transcription, followed by PCR amplification to generate overlapping double-stranded DNA amplicons covering each of eight segments of the influenza virus genome.

### Phylogenetic analysis

Phylogenetic analysis of sequences contained six gene segments (PB2, PB1, PA, HA, NP, and NS) which were found in triple-reassortant swine influenza viruses circulating in pigs. The genes encoding neuraminidase (NA) and M protein (M) were most closely related to those in influenza A viruses circulating in swine populations.

## PREVENTION

Prevention and control measures for swine influenza are based on our understanding of seasonal human influenza[29] and consideration of potential modes of transmission.

### Prevention of pig to human transmission

The transmission from swine to human is believed to occur mainly in swine farms where farmers are in close contact with live pigs. The use of vaccines on swine to prevent their infection is a major method of limiting swine to human transmission. Risk factors that may contribute to swine-to-human transmission include smoking and not wearing gloves when working with sick animals.

### Prevention of human-to-human transmission

Influenza spreads between humans through coughing or sneezing and people touching something with the virus on it and then touching their own nose or mouth. This virus is not transmitted through food. In humans it is most contagious during the first 5 days of the illness although some people, most commonly children, can remain contagious for up to 10 days. Diagnosis can be made by sending a specimen, collected during the first 5 days for analysis. Recommendations to prevent spread of the virus among humans include using standard infection control against influenza. This includes frequent washing of hands with soap and water or with alcohol-based hand sanitizers, especially after being out in public. Experts agree that hand-washing can help prevent viral infections, including ordinary influenza and the swine flu virus. Influenza can spread in coughs or sneezes, but an increasing body of evidence shows small droplets containing the virus can linger on tabletops, telephones, and other surfaces and be transferred via the fingers to the mouth, nose, or eyes. Alcohol-based gel or foam hand sanitizers work well to destroy viruses and bacteria. Anyone with flu-like symptoms such as a sudden fever, cough, or muscle aches should stay away from work or public transportation and should contact a doctor to be tested. Social distancing is another tactic. It means staying away from other people who might be infected and can include avoiding large gatherings, spreading out a little at work, or perhaps staying home and lying low if an infection is spreading in a community. Public health and other responsible authorities have action plans which may request or require social distancing actions depending on the severity of the outbreak.

As of May 5, 2009, the CDC[12] has recommended that health care workers who provide direct care for patients with known or suspected swine influenza infection should observe contact and droplet precautions, including the use of gowns, gloves, eye protection, face masks, and fit-tested, disposable N95 respirators. In addition, patients with confirmed or suspected swine influenza infection should be placed in a single-patient room with the door kept closed and airborne-infection isolation rooms with negative-pressure handling should be used whenever an aerosol-generating procedure is being performed.[30]

## TREATMENT

### Oral drugs

Presently government of India recommends tamiflue as a drug of choice which is available at all government health bodies. Human influenza A is susceptible to both oseltamivir and zanamivir, two antiviral medications approved for the prevention and treatment of influenza in the United States. Two classes of antiviral medication are available for the treatment of seasonal human influenza: neuraminidase inhibitors (oseltamivir and zanamivir) and adamantanes (rimantadine and amantadine). During the 2008–2009 influenza season, almost all circulating human influenza A (H1N1) viruses in the United States were resistant to oseltamivir.[31] However, genetic and phenotypic analyses indicate that swine influenza is susceptible to oseltamivir and zanamivir but resistant to the adamantanes.[32] The FDA has issued an emergency-use authorization that approves the use of oseltamivir to treat influenza in infants under the age of 1 year (treatment that is normally approved for those 1 year of age or older) and for chemoprophylaxis in infants older than 3 months of age (chemoprophylaxis that is normally approved for children 1 year of age or older) [Table 1].[28]

**Table 1:** Swine-origin influenza antiviral medication dosing recommendations

Agent, group		Treatment	Chemoprophylaxis
Oseltamivir Adults		75 mg capsule twice/day for 5 days	75 mg capsule once/day
Children (age 12 months/older)	15 kg or less	60 mg per/day divided into two doses	30 mg once/day
	15-23 kg	90 mg/day divided into two doses	45 mg once/day
	24-40 kg	120 mg/day divided into two doses	60 mg once/day
	More than 40 kg	150 mg/day divided into two doses	75 mg once/day
Zanamivir Adults		Two 5 mg inhalations (10 mg total) twice/day	Two 5 mg inhalations (10 mg total) once/day
Children		Two 5 mg inhalations (10 mg total) twice/day, age 7 years or older)	Two 5 mg inhalations (10 mg total) once/day (age 5 years or older)

### Swine flue vaccine

It’s considered a major medical breakthrough - India’s first home made swine flu vaccine named Vaxiflu-S^33^, (0.5ml i.m) is the first indigenous influenza vaccine in India since Independence. Its side effects, experts say, are minor which include fever, aches, and mild soreness. However, one in a million could be exposed to the risk of the Guillain Barre Syndrome - a rare neurological disorder. This vaccine is mainly for those above the age of 18. Trials for use by children will start in the future. Another vaccine called ‘Nasovac’ is the first intra-nasal vaccine for swine flu, produced in Pune, India. Nasovac[34] is available for Rs. 158 per dose, which can be used for adults and children above three years. At present no side effects of the vaccine are known, but not indicated in pregnant woman and lactating mothers.

## CONCLUSION

Prevention and control measures for swine influenza are based on our understanding of seasonal human influenza and consideration of potential modes of transmission. Clinicians are advised to see the H1N1 Influenza Center (NEJM.org) and the CDC Website (www.cdc.gov/h1n1flu/">www.cdc.gov/h1n1flu/">www.cdc.gov/h1n1flu/) for changes in guidance for testing, treatment, and infection control.
